# Multiband Spectrum Sensing Based on the Sample Entropy

**DOI:** 10.3390/e24030411

**Published:** 2022-03-15

**Authors:** Yanqueleth Molina-Tenorio, Alfonso Prieto-Guerrero, Rafael Aguilar-Gonzalez

**Affiliations:** 1 Information Science and Technology, Metropolitan Autonomous University Iztapalapa, Mexico City 09360, Mexico; yanqueleth@xanum.uam.mx; 2 Electrical Engineering Department, Metropolitan Autonomous University Iztapalapa, Mexico City 09360, Mexico; 3 Faculty of Sciences, Autonomous University of San Luis Potosi, San Luis Potosi 78210, Mexico; rafael.aguilar@uaslp.mx

**Keywords:** cognitive radio, sample entropy, software-defined radios, multiband spectrum sensing, real-time spectrum sensing

## Abstract

Cognitive radios represent a real alternative to the scarcity of the radio spectrum. One of the primary tasks of these radios is the detection of possible gaps in a given bandwidth used by licensed users (called also primary users). This task, called spectrum sensing, requires high precision in determining these gaps, maximizing the probability of detection. The design of spectrum sensing algorithms also requires innovative hardware and software solutions for real-time implementations. In this work, a technique to determine possible primary users’ transmissions in a wide frequency interval (multiband spectrum sensing) from the perspective of cognitive radios is presented. The proposal is implemented in a real wireless communications environment using low-cost hardware considering the sample entropy as a decision rule. To validate its feasibility for real-time implementation, a simulated scenario was first tested. Simulation and real-time implementations results were compared with the Higuchi fractal dimension as a decision rule. The encouraging results show that sample entropy correctly detects noise or a possible primary user transmission, with a probability of success around 0.99, and the number of samples with errors at the start and end of frequency edges of transmissions is, on average, only 12 samples.

## 1. Introduction

The concept of cognitive radio (CR) was introduced by Mitola and Maguire some years ago. It is described as the ability of one communications device to modify its parameters (such as modulation, carrier, bandwidth, etc.) to continue transmitting and/or receiving information [1]. Based on the fact that national regulatory administrations of the radio space attribute exclusive use licenses to transmit on fixed frequencies, two main protagonists are introduced by this concept: (i) the primary user (PU), also known as a licensed user, who is the user authorized to use a fixed frequency; (ii) the non-licensed user, also called secondary user (SU). The latter is the user that transmits or receives, dynamically taking advantage of the *spectral gaps*, which are the instants of time where the PU is not transmitting (or receiving), generating a *hole* in the spectrum [2].

Specifically, the operation of a CR is to select the best available channel, coordinate its access and, at the right time, leave the channel when it detects PU activity to avoid interference. This operation results in four functions or stages: spectrum sensing (SS), decision, sharing, and mobility [3]. SS is the ability to detect PU transmission in the spectral band that a SU is using or intends to use in a timely manner. The decision concerns the SU’s ability to select the best available spectrum band. Sharing refers to coordinated access to the channel selected by the SU, and spectrum mobility is the ability of a CR to vacate the used channel and to move to another possible available spectral gap when a PU is detected [2,3].

Considering the relevance of detecting a PU, the SS stage becomes probably one of the most important tasks in CRs. SS is performed considering characteristics of the spectral environment such as bandwidth, carrier frequency, or type modulation of the PU transmission [4]. This task, which usually is performed in one spectral band, can be carried out in several bands. The last environment is called multiband spectrum sensing (MBSS), which means that CRs can sense a wider frequency range where different bands are included, possibly involving several technologies [4]. Since future wireless communications services will require high performance, demanding among other things a higher bandwidth, and in real situations the available spectrum spaces are at non-contiguous frequencies, then it is necessary to have a broad overview of PU activity [4]. Here, there is a need to consider MBSS to perform the detection of available spectral spaces sweeping a wide bandwidth [5]. In this context, this work presents a MBSS method with a decision rule based on the sample entropy (SampEn). Frequency borders detection, the elimination of impulsive noise using the multiresolution analysis (MRA), and the Higuchi fractal dimension (HFD) as a decision rule were developed by the authors in previous works [6,7]. In these previous works, simulated and real-time controlled scenarios were implemented, obtaining outstanding results in terms of a detection probability varying the signal-to-noise ratio (SNR, defined as the ratio between the power of the signal that is transmitted and the power of the noise that corrupts it [8]). Now, to continue improving the performance, this decision rule is modified.

Entropy measures have been used before in SS, mostly in single-band detection. In [9], the approximate entropy (ApEn), bispectral entropy (BispEn), SampEn, and Rényi entropy (RenyiEn) were used in single-band SS. In this work, results show that BispEn outperformed the other three entropy measures, improving the detection by at least 5 dB. In [10], a technique based on the ApEn, with unknown levels of SNR in a single band, shows a favorable detection considering the signal cyclostationarity. In [11], a proposal is developed for the detection based on the entropy of the received signal, using appropriately matched filtering. Another work that has been published recently is [12], in which the authors propose a blind signal detector based on entropy to sense a sub-band of the power spectrum. In [13], a system for mobile communications using a software-defined radio network is implemented. Here, to detect PUs, RenyiEn is used directly on the spectra of the received signals without prior knowledge of their characteristics.

In this proposal, a decision rule is implemented using the SampEn. In this case, the authors are exploring the SampEn instead of the Higuchi fractal dimension (implemented before by the authors in [6,7]). Both methods are looking for similarities between the measured spectra and a PU transmission, discerning from a noise signal (usually, an additive Gaussian noise, AWGN). However, the SampEn has not been studied under a multiband scenario. Besides, the SampEn is considered to have a lower computational complexity for easy implementation in a real-time scenario. It is important to mention that in [6,7], the algorithms developed to detect the edges of the frequency intervals used by PUs and the noise inhibition due to the electronic devices used by USs are maintained given their excellent performance, as shown before. However, the decision rule, together with the detection of frequency edges, represents the other key part in MBSS. For this reason, changing it in order to improve the overall performance of the system is essential to obtain the best results, especially in real-time scenarios. This is the reason for the introduction of a new decision rule based on the SampEn.

This article is organized as follows. In Section 2, the SampEn is introduced. Section 3 describes the methodology used for all simulations and experiments. In Section 4, the simulation and implementation of the controlled environment are developed, followed by the presentation of the obtained results in Section 5. Finally, the conclusions are presented in Section 6.

## 2. Sample Entropy

SampEn was initially introduced by Richman and Moorman [14] to evaluate the complexity of the physiological time series and diagnose diseased states. The SampEn is obtained by the following steps.

**Figure 1:**
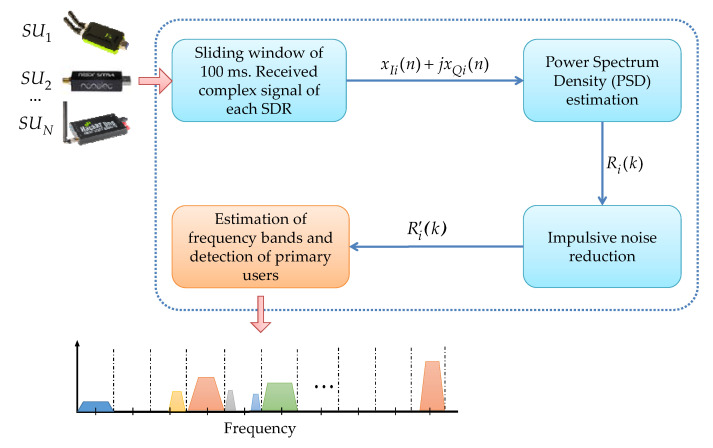
General scheme of implemented original MBSS scenario [7].

Considering the sequence $X=[x_{1},\ldots ,x_{K}]$ of length *K* (in our case, this sequence of samples represents each spectral frame, obtained from each one of the used devices or SUs shown in Figure 1), perform the following.

1. Build a vector $V_{P}$ with *m* consecutive data points taken from *X*; i.e.,
  (1) $$V_{P}=[x_{P},\ldots ,x_{P+m-1}]$$
  where *P* varies in the interval (1≤P≤K−m), and *m* is the length of sequences to be compared, also called the *embedding dimension*.
2. For each *P*, define
  (2) $$C_{P}^{m}\;=\;\frac{1}{K-m-1}\sum_{h=1,\;h\neq P}^{K-m}\Theta \left(r-{\left\|V_{P}-V_{h}\right\|}_{1}\right)$$
  where r is the tolerance for accepting matches, and it is usually selected as a factor of the standard deviation of vector X, std(X). Θ(   ) is the Heaviside function
  (3) $$\Theta (X)\;=\;\{\begin{array}{c} 0,\;\;\;X<0\\ 1,\;\;\;X\geq 0 \end{array}$$
  and ${\left\|\right\|}_{1}$ is the Chebyshev distance, defined as
  (4) $${\left\|V_{P}\;-\;V_{h}\right\|}_{1}=\max\left(\left|x_{P}-x_{h}\right|,\ldots ,\left|x_{P+m-1}-x_{h+m-1}\right|\right)$$
  where $C_{P}^{m}$ represents the proportion of $V_{h}$, *P* ≠ *h* whose distances to $V_{P}$ are less than *r*.
3. Now, for each *P*, define
  (5) $$C_{P}^{m+1}\;=\;\frac{1}{K-m-1}\sum_{h=1,\;h\neq P}^{K-m}\Theta \left(r-{\left\|V_{P}-V_{h}\right\|}_{1}\right)$$
  where $C_{P}^{m+1}$ represents the proportion corresponding to the dimension of *m* + 1. $C_{P}^{m}$ and $C_{P}^{m+1}$ have the same mold, but embedding vectors in both cases are defined in different spaces.
4. Average across all embedding vectors to obtain
  (6) $$U^{m}\;=\;\frac{1}{K-m}\sum_{P=1}^{K-m}C_{P}^{m}$$
  and
  (7) $$U^{m+1}\;=\;\frac{1}{K-m}\sum_{P=1}^{K-m}C_{P}^{m+1}$$
5. The SampEn is computed as
  (8) $$SampEn\;=\;-\ln\left(\frac{U^{m+1}}{U^{m}}\right)$$

SampEn represents the negative natural logarithm of the conditional probability that two similar sequences for *m* points remain similar at the next point, where proper matches are not included in the probability calculation. Therefore, a lower value of SampEn indicates more self-similarity of the studied sequence, while a higher value of SampEn indicates a greater complexity of the sequence. 

The calculation of SampEn requires the a priori determination of two unknown parameters: *m* and *r* (the length *K* of the data, as mentioned before, depends on the dynamic size window that corresponds to a section of the power spectrum density (PSD) of each connected device). Suggested values of *r* are in the interval of [0.1, 0.2] times the standard deviation of the processed signal *X*. The choice of a correct *r* is not an easy task. Indeed, in [15], it was studied how to fit to the data the best *r* value for the specific case of the ApEn (a similar entropy of the SampEn) evaluating all the values of *r* varying from 0 to 1 looking for the maximum of the ApEn (considered as the correct interpretation of the complexity of a signal). It was concluded that the maximum of ApEn does not always occur within the prescribed interval of *r* values. Hence, computing all possible *r* values is impractical due to the computational burden this represents. For this, practical recommendations with good results, given for other applications, are in the interval [0.1, 0.2]. Based in this fact (and leaving the search for the optimal value of *r* for our analyzed data as an open research topic), in our simulations, we set r=0.1. The value of *m* can be calculated by estimating the false nearest neighbor [16]. In this MBSS technique, the m found across false nearest neighbors for our signals was most of the time equal to 2. Therefore, it was set at m=2 for all simulations and experimental tests. SampEn is, theoretically speaking, a fraction on the interval 0<SampEn<∞. However, the following two formulas can be used to find the lower and upper limits of SampEn for fixed values of *m* and *K*.

The lower limit can be estimated as

(9) $$2{\left[\left(K-m-1\right)\left(K-m\right)\right]}^{-1}$$

and the upper one as

(10) ln(K−m)+ln(K−m−1)−ln(2)

## 3. Methodology

The main idea of this work is to show that SampEn is a viable technique to be used as a decision rule to distinguish noise or a possible PU transmission in a real-time MBSS scenario. The original scenario developed by the authors in [7], using software-defined radios (SDR) devices such as SDR-LTR, HackRF One, and LimeSDR mini, configured to work as SU and PU, is the base for this work. A brief description of different modules integrating this base methodology, shown in Figure 1, is given below.

1. **Sliding time window.** This block receives the complex signal from each connected device i=1,…,N, refreshed every 100 ms. In this block, it is possible to manipulate the parameters of the sampling rate $f_{Si}$, center frequency $f_{Ci}$, and gain of each device *i*.
2. **Estimation of the power spectral density.** In this block, from the signal $x_{Ii}\left(n\right)+jx_{Qi}\left(n\right)$, the PSD is computed directly in a linear scale for every frame of 100 ms. For this, a Welch estimator [17] is used, resulting in the signal $R_{i}\left(K\right)$. The number of samples for each frame, used to estimate PSD, is a value chosen by the user and depends on the type of used device: 512, 1024, 2048, or 4096 for the RTL-SDR devices, and 1024, 2048, 4096, or 8192 for HackRf One and LimeSDR mini devices.
3. **Impulsive noise inhibition.** Impulsive noise and high-frequency noise are greatly reduced in this module. Here, the processing is done through the detail coefficients and the approximation coefficients resulting from having applied the MRA to the PSD estimate [18]. This noise reduction proposal is a novel technique introduced by the authors, giving excellent results. The result of this block is the modified PSD ${R^{\prime }}_{i}$.
4. **PU detection.** This module detects spectral gaps and possible PU transmissions in a wide frequency range. As can be seen in [6], MRA and *K*-means [19] algorithms are used to detect the start and end edges of a PU transmission, and HFD [20] is used as the decision rule to distinguish what is noise from a possible transmission.

**Figure 2:**
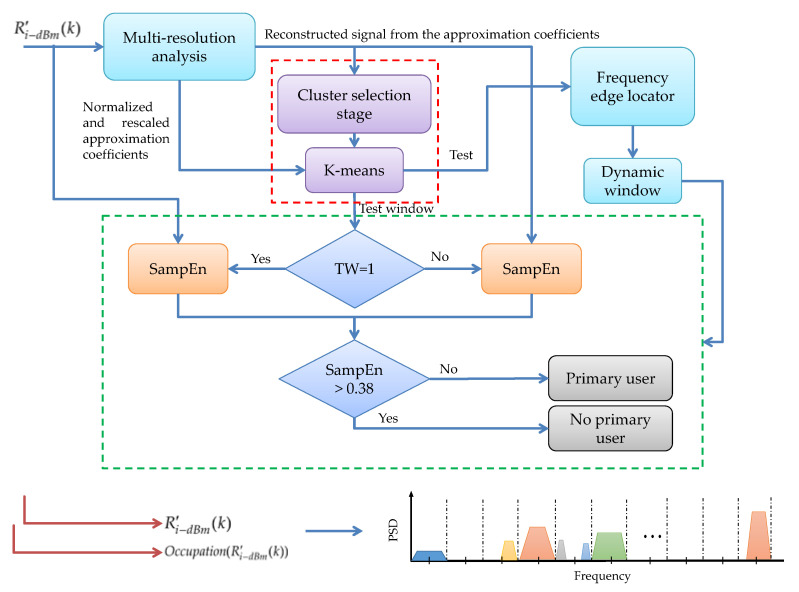
Modified module for PU detection using the SampEn.

In this work, some basic steps of the original methodology are retaken. The current contribution appears in the PU detection block (colored in orange in Figure 1). Figure 2 shows in detail this modified module, in which the SampEn is included as a decision rule. This module is described in the next points.

1. PSD perceived by each connected SDR device is evaluated in dBm to obtain the ${R^{\prime }}_{i-dBm}\left(K\right)$ signal. MRA is applied to this signal, resulting in the approximation and detail coefficients at different levels of decomposition.
2. The analysis of the reconstructed signal of the approximation coefficients allows us to know the number of clusters in which the normalized and rescaled approximation coefficients will be classified by the *K*-means algorithm to build the *test signal*.
3. Processing the changes of state of this signal, it is possible to detect the start and end edges of the transmissions in the analyzed frame. With this information, it is possible to build windows of dynamic size.
4. Each dynamic window (*test window*) represents a section of the spectrum that corresponds directly to a section of the *test signal*; i.e., to a section of the reconstructed signal from the coefficients. Each *test window* is compared with a threshold equal to 1. If the *test window* comparison matches the threshold, the SampEn will be directly applied to the original spectrum window. Otherwise, the SampEn will be applied to the reconstructed spectrum window.
5. If the value of the SampEn is greater than 0.38, it means only noise is present in the analyzed window. If the value of the SampEn is lower, it is *highly* probable that there is a PU in the analyzed window. Finally, processing each frame of each connected SDR device, the *occupation* is obtained; i.e., the spectral location of the PU and the possible spectral gaps in which the SU could be placed. In Section 5.1, the choice of the decision threshold is justified.

## 4. Simulations and Real-Time Controlled Scenario

In this section, simulations and experimental tests are presented. To evaluate this method, two different schemes are proposed: a simulation considering synthetic signals (explained in Section 4.1) and a real-time controlled environment using SDR devices as transmitters and/or receivers (explained in Section 4.2).

Before detailing the implemented scenarios, it is necessary to indicate the inclusion of the noise in both simulation and real-time implementation in order to evaluate the method’s performance as a function of SNR.

**Figure 3:**
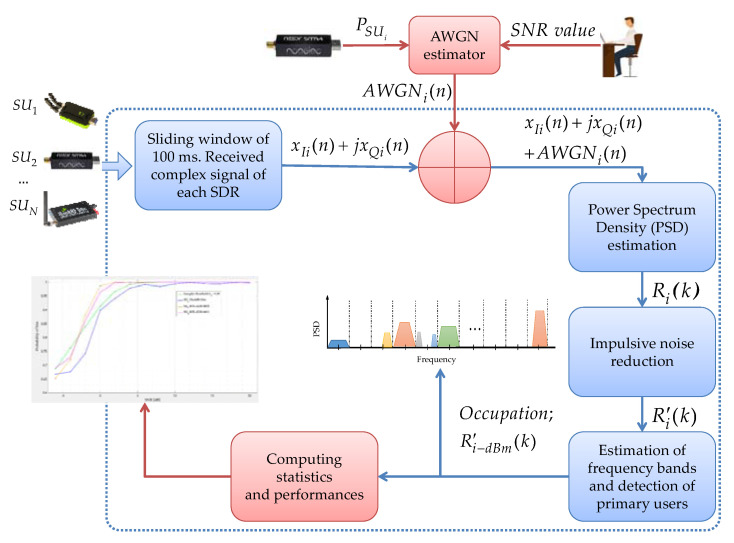
Steps for simulation and controlled implementation.

For this, we added a white noise process with a Gaussian distribution (AWGN) to each received signal in time, modifying its power to get a specific SNR; i.e., the $AWGN_{i}$ added to each $x_{Ii}\left(n\right)+jx_{Qi}\left(n\right)$ signal has the function of computing (i) the SNR value that is desired in the controlled scenario and (ii) the obtained power $P_{SU_{i}}$ from each connected SU. This addition is shown in Figure 3.

### 4.1. Simulation

The simulation was carried out considering a PU randomly appearing in a wide band. For this, the scenario was implemented in the MATLAB software with the parameters defined in Table 1. The performance of SampEn was studied by applying it to sequences of points that correspond to transmissions with AWGN—specifically, orthogonal frequency division multiplexing (OFDM) symbols. This simulation is necessary to corroborate the better performance of entropy than the HFD before delving into its real application. Results of this simulation are presented in Section 5. 

### 4.2. Implementation of the Controlled Scenario

This section details the implementation of the methodology in a controlled wireless communication environment. For this, the SDR devices described in Table 2 were used. The versatility of software-defined radios foresees its possible application in the improvement of short-wave communications [21,22,23]; for example, to facilitate spectrum examination, interference detection, efficient frequency distribution assignment, the testing of the operation of repeater systems and the measurement of their electrical parameters, the identification of spectrum intruders, and the characterization of noise in different spectral bands. Furthermore, linked to these main characteristics and their relatively low cost, SDR devices have the flexibility given by programming their features with free software, achieving multiple possibilities such as the construction of transmitters, receivers, or multiplexers [24,25].

**Figure 4:**
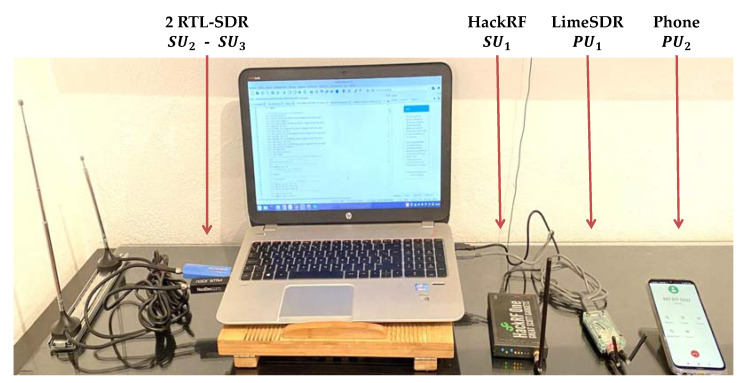
Controlled short-distance wireless communication environment [7].

Based on the advantages offered by the SDR devices mentioned above, it is proposed to use them in the real-time scenario of short-distance wireless communications, as shown in Figure 4. The goal of this scenario, proposed by the authors in [7], is to have a point of comparison between the SampEn and the Higuchi fractal dimension, previously tested for MBSS. The scenario includes three SUs and two PUs, covering a wide band. These users are positioned at the central frequencies indicated in Table 3.

This controlled scenario was implemented so that the comparison between both techniques was as realistic as possible. However, given that we have a real communication environment, there is an important point to consider: it is almost impossible to isolate the external transmissions that can influence the result, including a classical external additive noise affecting communications (this is different from the controlled noise added in the received signals to get a desirable SNR for performance evaluation purposes).

## 5. Results

In this section, results obtained regarding the probability of success and the number of samples in error for bot, simulated and experimental tests are presented. However, to carry out both tests, it is first necessary to set a critical value: the threshold for the sample entropy in the decision rule. In the next paragraphs, this choice is discussed.

### 5.1. Threshold Selection in the Decision Rule

**Figure 5:**
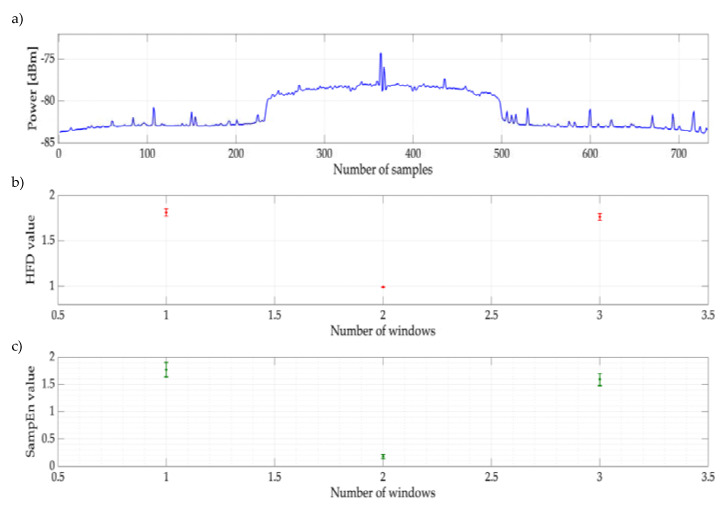
(**a**) Example of a frame corresponding to an OFDM transmission with AWGN (SNR = 10 dB). (**b**) Result of applying the HFD over 1000 frames. (**c**) Result of applying the SampEn over 1000 frames.

Before establishing the threshold in the decision rule and understanding how the proposed methodology works, we present an example in which the SampEn was basically applied to two different sequences of points: a primary transmission (OFDM symbol) and an AWGN. For this, the SampEn was applied to a frame divided into three sections (windows): two of these sections correspond to AWGN and one to an OFDM transmission. The complete frame, shown in Figure 5a, is integrated by samples (number) 1 to 225 and 500 to 743 corresponding to the AWGN and samples 226 to 499 containing the OFDM transmission. This process was repeated for 1000 frames (or random realizations) with a specific SNR value of 10 dB. The result (average and standard deviation) of applying the SampEn to these frames is shown in Figure 5c. In parallel, the HFD (used as a decision rule in previous works and just for comparison purposes) was applied to these same frames, and its results are shown in Figure 5b. Clearly, SampEn (as HFD also does) differentiates what is noise and what is a transmission. We can observe that the SampEn is close to 0 when the analyzed sequences have great similarity with themselves (in this case, a possible transmission of the PU). On the contrary, the SampEn tends to a value equal to 2 or higher when the discrete series is more complex (in this case, the AWGN). 

**Figure 6:**
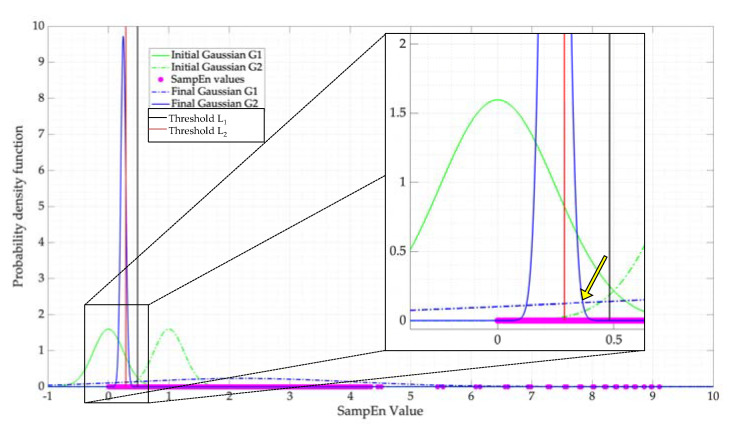
EM algorithm applied to the values of the SampEn to determine the optimal decision threshold.

Now, knowing that SampEn permits us to classify these two states, a question needs to be answered: what is the optimal threshold to classify both states considering the SNR influence? For this, the blind classification Expectation–Maximization (EM) algorithm was implemented [31]. Based on the kind of processed signals, it is feasible to think that the point where the two Gaussians intersect is the optimal SampEn threshold. In this way, 2,084,015 values of the SampEn were analyzed by EM with SNR values ranging from −6 to 20 dB. Figure 6 shows the result of applying EM to the values obtained from this process. The values $L_{1}=\mu_{1}+n\sigma_{1}$ and $L_{2}=\mu_{2}+n\sigma_{2}$ with n=0.5 are used to delimit the region in which a threshold can be chosen. This region is relatively close to the intersection of both Gaussians ($L_{o}=0.38$). Besides, these limits ($L_{1}$ and $L_{2}$) are proposed as lower and upper limits, respectively, to compare the result of taking specific values for SampEn.

### 5.2. Simulation Results

Performance evaluations for both experimental and simulated tests are based on the probability of success (PS), which is the result of counting the total of correctly located frequency windows with respect to the total number of detected frequency windows.

**Figure 7:**
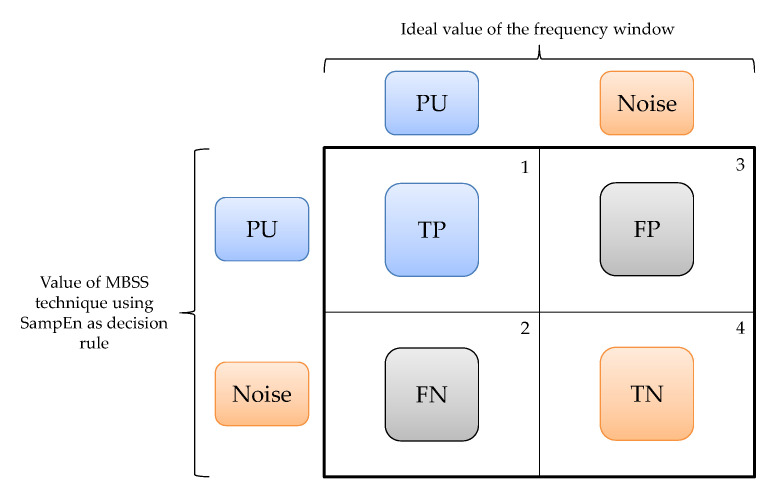
Evaluation outcomes of detected windows with the proposed MBSS technique.

To determine this parameter (i.e., PS), four possible cases are considered (see Figure 7):

- A window that corresponds to a PU transmission and that SU classifies as a PU transmission is a true positive (TP) value.
- A frequency window that corresponds to a transmission of the PU and that SU classifies as noise is a false negative (FN) value.
- A window that corresponds to noise and that SU classifies as a PU transmission is a false positive (FP) value.
- A frequency window that corresponds to noise and that SU classifies as noise is a true negative (TN) value.

PS is evaluated as

(11) $$PS=\frac{TP+TN}{TP+FP+FN+TN}$$

**Figure 8:**
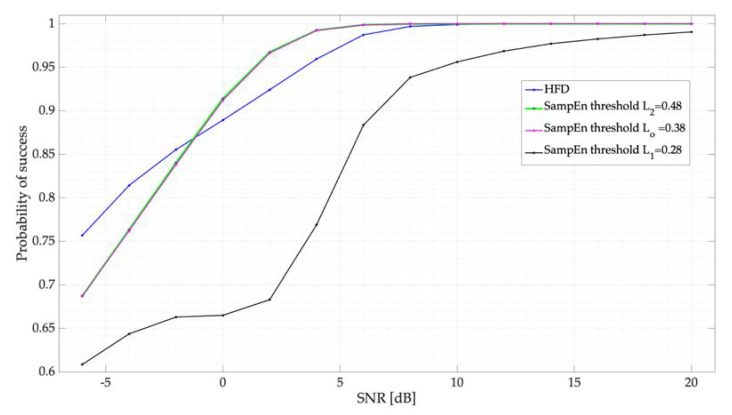
Probability of success for different proposed thresholds for the SampEn. Results are compared with the HFD.

Results of the simulated test using the proposed thresholds, mentioned in Section 5.1, are displayed in Figure 8. Clearly, there is no difference in choosing between the optimal threshold *L_0_* = 0.38 and the upper bound 0.48, with both giving the same results. Threshold values higher than 0.48 have less efficiency in the proposed system. Figure 8 shows both the results obtained by the SampEn and the HFD considering, as mentioned in Section 4.1, only one simulated PU per frame. Both techniques clearly show an excellent performance for SNR values greater than or equal to 0 dB. For lower values of SNR, both techniques show a similar, good performance. 

**Figure 9:**
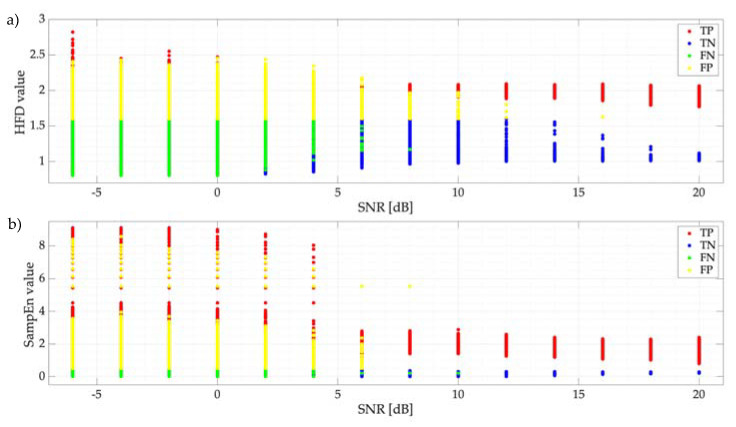
(**a**) Classification of detected windows using (**a**) the HFD and (**b**) SampEn (threshold set at 0.38) as decision rules.

Figure 9 shows the classification of the detected windows according to Figure 7. Figure 9a shows this classification when data are analyzed with the HFD, and Figure 9b shows those with the SampEn. Here, a clear difference is shown between these two decision rules when the SNR tends to negative values. Indeed, as the SNR decreases, the value of the SampEn and the HFD begin to increase due to the complexity of the signal, and the algorithm begins to misclassify the different dynamic size windows.

**Figure 10:**
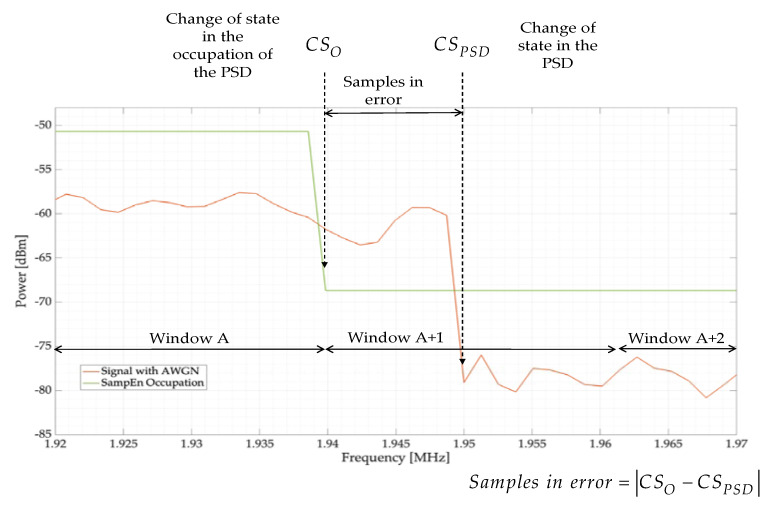
Graphical description of what the samples in error, regarding the dynamic window and occupation, represent.

Another parameter to evaluate the proposed methodology is the samples in error; i.e., the number of samples that exist between the ideal value of the frequency edge of the controlled transmission and the detected point at which the proposed method locates the PU transmission (see Figure 10). This figure also shows the dynamic window concept, which is the number of blocks in which the PSD is sectioned, and the occupation of the PSD, which is the union of the results obtained for each window.

**Figure 11:**
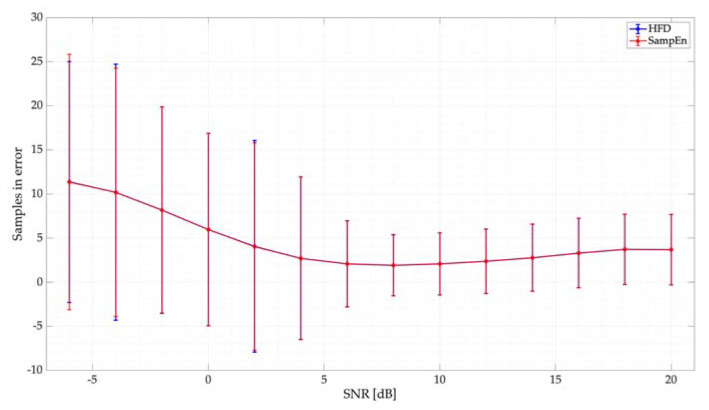
Samples in error for the proposed threshold for the SampEn. Results are compared with the HFD.

Figure 11 shows the samples in error for the simulated tests. Here, the behavior of both the HFD and SampEn, with a threshold $L_{o}=0.38$, is practically the same: an excellent detection for SNRs higher than 5 dB (less than 5 samples in error on average) and a very good detection for lower SNRs (on average, only 10 samples in error). In this case, the samples in error, on average, represent 0.67% of the frame evaluated in this section.

### 5.3. Results of the Controlled Scenario

**Figure 12:**
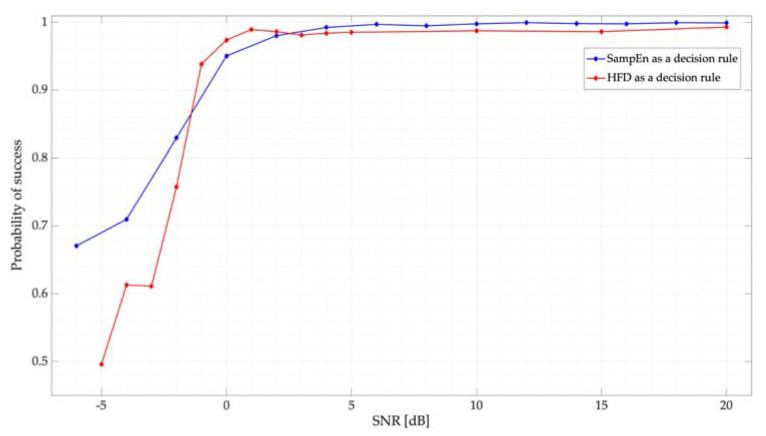
Probability of success in terms of the SNR of the real controlled environment.

The results, in terms of PS and SNR, of the implementation of the controlled wireless communication environment are presented in Figure 12. Here, the average results of the method’s efficiency at detecting the three connected PUs using the HFD and the SampEn are displayed. The behavior presented by both techniques to correctly determine the presence of PUs in the sensed spectral interval is practically the same. Both techniques have an excellent performance for SNR values greater than 0 dB, at around 0.98 for the HFD and 0.99 for the SampEn. For SNR values less than 0 dB, the HFD shows an abrupt decay. On the other hand, SampEn keeps very good results, even in very noisy cases. 

**Figure 13:**
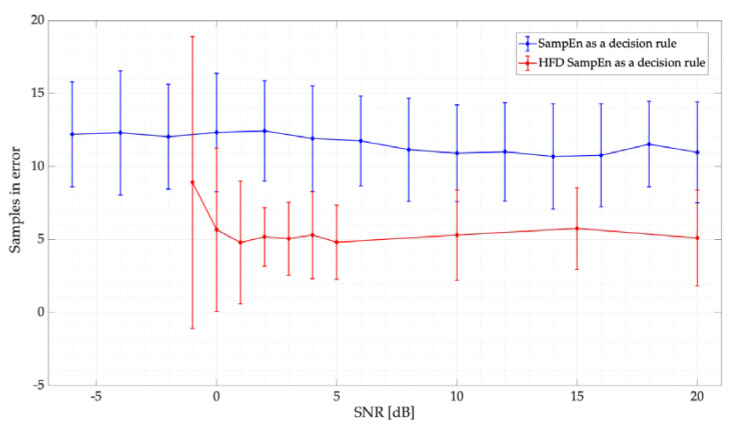
Samples in error vs. SNR for the proposed real-time implementation.

In Figure 13, the results (mean and standard deviation) of the samples in error for this real scenario considering three different PUs are also presented. We can observe that, in this case, the HFD slightly outperforms the SampEn. However, the number of samples in error is, in general, relatively small for both techniques showing an excellent performance in terms of accurately locating PUs in the wide spectrum. In this case, the samples in error on average for the SampEn amount to 0.58% for $SU_{1}$ and 1.61% for $SU_{2}$ and $SU_{3}$ of the evaluated frame. On the other hand, for the HFD, the erroneous samples amount to 0.29% for $SU_{1}$ and 0.8% for $SU_{2}$ and $SU_{3}$.

**Figure 14:**
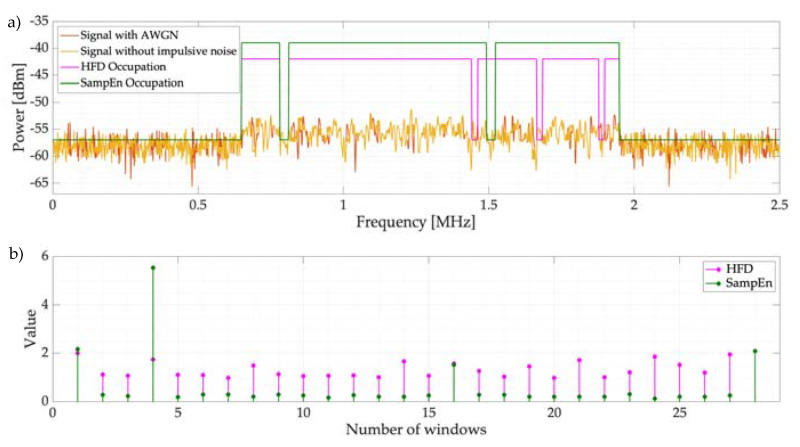
(**a**) Occupation of an example frame using HFD and SampEn with a low SNR. (**b**) Computed value of HFD and SampEn for each detected window in the spectrum frame.

Finally, a specific challenging case (i.e., an explicit frame) is shown for both techniques: the HFD and SampEn. In this frame, there is a PU with an OFDM transmission with a bandwidth of 1 MHz (Figure 14a). This analyzed frame has an SNR value of 0 dB, which means that the signal is practically embedded in the noise. As a result of applying the proposed algorithm to this frame, the *pccupation* is displayed in Figure 14a as a continuous signal containing the changes of state: occupied and not occupied. Figure 14b shows the ordered number of windows in which the spectrum is segmented and their respective values computed with the proposed methodology for both techniques—SampEn and HFD. The number of windows is the same for both techniques because they use the same edge detector to form the different windows of dynamic size. In this example, both methodologies detect the PU; however, other spectrum holes in error are also detected. Hence, we identify an important issue: when the SNR is reduced, an increasing number of dynamic windows will form. This means that the algorithm needs to process increasing numbers of windows, making the methodology inefficient from the point of view of computational resources.

## 6. Conclusions

In this work, a novel MBSS technique based on the sample entropy is presented. For this, a low-cost SDR devices-based system is implemented to measure its performance in a real wireless communications environment at detecting primary users in a wide frequency domain. From the results, some important points could be highlighted:

- Experimental results for the detection of the primary users are consistent with the results of the simulated calculation, indicating that the proposed MBSS technique is correct and feasible to be considered for a real wireless communication environment. Power levels of the proposal are like those where spectrum sensing can be considered; for example, the case of coexistence between LTE-U and Wi-Fi [32].
- It is difficult to give an interval in which the SampEn is located—this is because, in this investigation, the frames analyzed by the proposed technique are variable, and these can change depending on the SNR or the number of samples that the PSD contains. However, it is possible to observe (see Figure 9) that the SampEn value is between 0 and 9. These limits can be obtained with Equations (9) and (10). Nevertheless, the value obtained for these edges is not exact in a real environment since the number of samples of each analyzed window is variable. On the other hand, for an ideal environment in which the frame analyzed for both noise and a PU transmission is 512 samples, the lower limit is $7.7\times 10^{-6}$ and the upper limit is 11.77. Besides, it is possible to observe from this same figure that the entropy always increases as the SNR decreases.
- Results of applying $L_{2}$ or $L_{o}$ as the threshold for the SampEn are practically the same. Hence, the question arises: what is the maximum value that the SampEn could take while permitting good performance to be obtained in terms of classifying the two possible states? It is possible to consider this topic as a future research perspective.
- Both decision rules, HFD and SampEn, are fast and feasible techniques to detect the presence of a PU in the spectrum. From Figure 12, it is possible to see that both techniques have excellent performance in scenarios with SNR values greater than 0 dB; however, for values less than 0 dB, SampEn has better performance. With this analysis, it is possible to affirm that both techniques, which measure the complexity of a signal, are accurate in detecting PUs in a multiband environment.
- Finally, with an average of 99% precision at detecting the PU, and with 12 samples (mean) showing errors in locating the start and end edges of a transmission, we can conclude that SampEn is a viable technique for the precision detection of PUs in a wide spectrum.

## Figures and Tables

**Table 1:** MBSS simulation parameters.

Software	MATLAB 2019
SNR values	−6 to 20 dB spaced by 2 dB
Number of frames per each SNR value	10,000
Samples per frame	743
Number of symbols per frame	One symbol, OFDM

**Table 2:** SDR device characteristics [26] (MSPS, mega samples per second).

Device	HackRF One [27]	RTL-SDR [28]	LimeSDR Mini [29]
Frequency range	[1 MHz–6 GHz]	[22 MHz–2.2 GHz]	[10 MHz–3.5 GHz]
RF bandwidth	20 MHz	3.2 MHz	30.72 MHz
Sample depth	8 bit	8 bit	12 bit
Sample rate	20 MSPS	3.2 MSPS	30.72 MSPS
Tx channels	1	0	1
Rx channels	1	1	1
Duplex	Half	-	Full
Transmit power	−10 dBm + (15 dBm @ 2.4 GHz)	-	Max 10 dBm (depending on frequency)

**Table 3:** Characteristics of each user.

Tx/Rx	*SU*_1_	*SU*_2_	*SU*_3_	*PU*_1_	*PU*_2_
Device	HackRF One	RTL-SDR 0005	RTL-SDR 0002	LimeSDR Mini	Cell phone call
Tx Frequency (MHz)	-	-	-	847.8	842.5
Type of transmission	-	-	-	OFDM	CDMA [30]
Tx Bandwidth (MHz)	-	-	-	1	5
Rx Frequency (MHz)	835	846.2	848.6	-	-
Rx Bandwidth (MHz)	20	2.4	2.4	-	-

## Abbreviations

CR	Cognitive radio
PU	Primary user
SU	Secondary user
SS	Spectrum sensing
MBSS	Multi-band spectrum sensing
SampEn	Sample entropy
MRA	Multiresolution analysis
HFD	Higuchi fractal dimension
SNR	Signal-to-noise ratio
ApEn	Approximate entropy
BispEn	Bispectral entropy
RenyiEn	Rényi entropy
PSD	Power spectral density
EM	Expectation maximization
Tx	Transmitters
Rx	Receivers
MSPS	Mega samples per second
TP	True positive
FN	False negative
FP	False positive
TN	True negative
OFDM	Orthogonal frequency division multiplexing
AWGN	Additive white Gaussian noise
PS	Probability of success
